# Efficacy of a Mixture of Diosmin, Coumarin, and Arbutin on Preventing Breast Edema in Implant-Based Breast Reconstruction

**DOI:** 10.1093/asjof/ojaf090

**Published:** 2025-07-08

**Authors:** Riccardo Di Giuli, Camilla M E Bonzi, Silvio M Capuano, Stefano Vaccari, Flavio Bucci, Riccardo Fondrini, Marco Klinger, Francesco Klinger, Valeriano Vinci

## Abstract

**Background:**

Postmastectomy breast edema is a common complication in patients undergoing immediate implant-based reconstruction, leading to physical and psychological consequences. Despite its clinical relevance, standardized prophylactic strategies remain lacking.

**Objectives:**

The authors of this study aim to evaluate the efficacy of an anti-edema formulation containing diosmin, coumarin, and arbutin (Linfadren, Omega Pharma Srl, Italy) in reducing postmastectomy breast edema and improving postoperative outcomes.

**Methods:**

A prospective, randomized clinical trial was conducted at a single tertiary care center. Women aged ≥18 years undergoing mastectomy with immediate implant-based reconstruction were randomized (1:1) into 2 groups: control (standard postoperative prophylaxis) and experimental (standard prophylaxis plus Linfadren, Omega Pharma Srl). Mastectomy flap thickness was measured using Vernier calipers, and breast edema symptoms were assessed with the Breast Edema Questionnaire (BrEQ). Follow-up visits were scheduled weekly during the first month, with additional evaluations at 6 weeks and 2 months.

**Results:**

The experimental group demonstrated a significant reduction in mastectomy flap thickness (45.95% decrease at 2 months vs stable or increased thickness in the control group, *P* < .05). BrEQ scores improved more rapidly in the experimental group, reflecting reduced symptoms of breast edema. Pitting edema prevalence was consistently lower in the experimental group compared with the control group throughout the study (*P* < .05).

**Conclusions:**

In this study, the authors highlight the potential of Linfadren (Omega Pharma Srl) as an effective prophylactic treatment for postmastectomy breast edema. By mitigating edema, this approach may improve postoperative recovery, reduce complications, and enhance patients’ quality of life. Further large-scale studies are warranted to validate these findings.

**Level of Evidence: 2 (Therapeutic):**

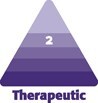

Breast edema, marked by fluid accumulation in breast tissue, often arises after breast cancer treatment and presents with swelling, heaviness, inflammation, skin thickening, and pain.^1,2^ Research on postoperative breast edema remains limited, and no standardized diagnostic or therapeutic approach is available. Frequently underestimated, it is perceived as an unharmful side effect that naturally resolves over time.^3^ Nonetheless, patients may contend with physical and psychological consequences, encompassing body image concerns, anxiety regarding cancer recurrence, breast discomfort, pain, and deterioration of quality of life.^1,3^

Lack of established diagnostic methods leads to delayed identification and symptoms progression, and proposed treatments such as antibiotics and pain medications, may only exacerbate symptoms.^1^

Although breast edema following breast-conserving surgery has been extensively studied, research on postmastectomy edema is lacking.^1-4^

The lack of a gold standard for detecting breast edema often leads to its neglect by physicians, resulting in patients suffering from various and potentially severe physical and psychological consequences.^5^ Because of the insufficient recognition of this condition, there is a general lack of understanding among both patients and physicians on how to adequately treat breast edema.^6^

This delay in diagnosis and treatment allows the condition to progress, worsening the symptoms and often leading to inadequate or palliative treatment measures, such as chronic use of antibiotics or pain medications. These measures not only fail to improve the edema but can also exacerbate the psychological and physical symptoms experienced by patients.^7^

Mastectomy is often associated with manipulation of lymphatic structures such as axillary dissection, increasing the risk of lymphatic stasis and damage, and constitutes a potent inflammatory trigger for breast tissues.^8^ Additionally, breast edema can predispose patients to complications like seroma, infection, implant removal, and overall reconstruction failure.^9-15^

In this study, the authors examine the prophylactic potential of an anti-edema compound combining coumarin, arbutin, and diosmin (Linfadren, Omega Pharma Srl, Italy) to prevent breast edema in mastectomy patients undergoing immediate implant-based reconstruction. Such a compound reduces edema by enhancing lymphatic drainage and microcirculation. Diosmin improves venous tone and reduces capillary permeability, coumarin promotes lymphatic flow, and arbutin has anti-inflammatory effects. Together, they help minimize postsurgical swelling and improve recovery. Linfadren is well-tolerated but may cause mild gastrointestinal discomfort, headache, dizziness, hepatic toxicity, or allergic reactions.^16^

## METHODS

This study was conducted according to Declaration of Helsinki, ICH-GCP, and CONSORT guidelines. Before enrollment, patients received a study overview and provided informed consent. Eligible participants were women aged 18 or older with breast cancer, undergoing unilateral or bilateral mastectomy with immediate implant-based reconstruction. No acellular dermal matrices were used. The only exclusion criterion was nonadherence to the treatment regimen.

Mastectomy and reconstruction procedures were performed by 2 senior surgeons. When a nipple-sparing mastectomy was performed, we always preferred an italic-S incision in the outer quadrants. Patients were randomized 1:1 to receive either standard postoperative care or standard care plus an anti-edema formulation. The control group received an elastocompressive dressing for 7 days, a supportive bra, pain management with paracetamol, and selective antibiotic prophylaxis. The experimental group received these treatments plus Linfadren (Omega Pharma Srl; a combination of coumarin, arbutin, and diosmin) to reduce breast edema. No side effects were reported by the experimental group. Paracetamol was recommended for pain or fever management. Antibiotic prophylaxis with cefalexin monohydrate (1000 mg, twice daily until drainage removal) was administered in specific cases, such as when patients had significant comorbidities (eg, diabetes), an immunocompromised state, or a history of chemoradiation therapy. Throughout the follow-up period, patients participated in regular physiotherapy sessions aimed at enhancing ROM. Scar massage therapy was typically performed ∼4 to 5 weeks after surgery, unless complete wound healing had not yet been achieved.

Patients in the experimental group received the same postoperative prophylaxis as the control group, in addition to an anti-edema medication based on a combination of coumarin, arbutin, and diosmin (Linfadren), to reduce breast edema formation. Such therapy was started on the first postoperative day, taken on an empty stomach, twice a day for 2 weeks, followed by once daily for 4 weeks. Medication adherence was fostered by instructing patients to keep a daily diary, and they were reminded to take the medication during each outpatient visit and physiotherapy session over the initial 4 weeks of treatment, and patients were periodically contacted by phone to assess therapy adherence.^17^

### Outcome Measures

Follow-up lasted 2 months, consisting of weekly visits during the first month, a 6-week checkup, and a final evaluation 2 months after breast reconstruction. At each visit, alongside a comprehensive clinical assessment, patients were required to complete a Breast Edema Questionnaire (BrEQ).

**Figure 1.**
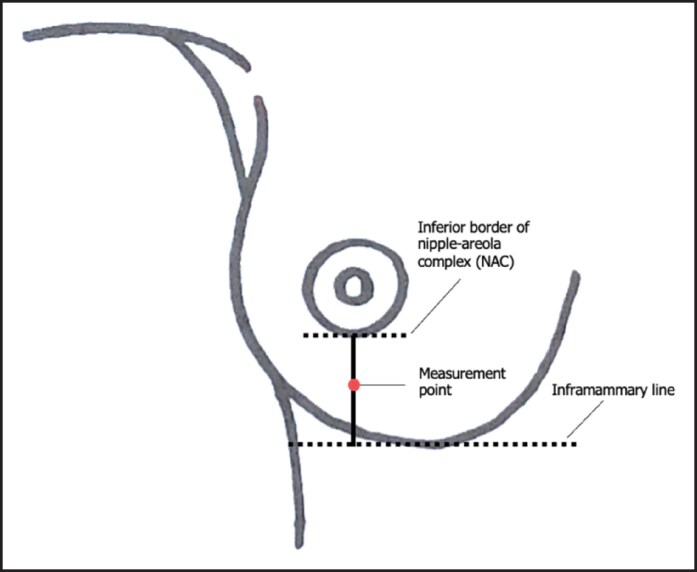
Measurement point for mastectomy flap thickness.

The primary objective of this study was the quantitative assessment of mastectomy flap edema thickness. Baseline measurements were acquired after 1 week of treatment, during the initial postoperative ambulatory visit. The thickness was measured at the inferior pole of the breast employing a standard low-pressure Vernier caliper.^18,19^ After applying a consistent pressure for 20 s, the imprint measurements would provide a direct correlation to the flap edema and would serve as an indirect measure of flap thickness. Measurements were taken from the midpoint of an imaginary line connecting the inframammary fold to the center of the inferior border of the nipple–areola complex (NAC; Figure 1). In cases of total mastectomy, the midpoint of the mastectomy scar was utilized as a reference point in lieu of the NAC. Measurements were taken on patients in a supine position with the head of the bed inclined at a 30° angle. To address the concern regarding potential bias, we have ensured that all measurements were consistently taken by the same researcher (medical doctor). Additionally, to enhance accuracy, each measurement was conducted twice, and the average of these measurements was used in our analysis.

The secondary outcome measure encompassed the evaluation of symptoms associated with breast edema. An Italian-translated BrEQ was selected as the assessment instrument.^4^ The questionnaire examines 8 core symptoms using an 11-point Likert scale. Individual item scores were then aggregated to derive a total symptom score. A predetermined threshold of ≥8.5 was employed to distinguish patients with breast edema from those without.^4^ Patients were instructed to complete the questionnaire based on their symptoms at the time of the visit.

Additionally, the presence of pitting edema was assessed in each breast quadrant by a second independent operator. This assessment involved the application of a constant pressure with 1 finger to the breast for 10 s. Upon finger removal, pitting edema was considered positive if indentation of the skin was observed in at least 1 breast quadrants.

### Statistical Analysis

Demographic and surgical data were subjected to comprehensive analysis. Potential confounding variables, including comorbidities and factors such as smoking, chemotherapy, radiotherapy, and axillary dissection, were evaluated.

Paired-sample *t* tests were applied to assess variations in mastectomy flap measurements and BrEQ results within each study group. A comparison was made between the outcomes at baseline (1 week following surgery), at 6 weeks (the end of the treatment period for the experimental group), and at 2 months follow-up. Independent samples *t* tests were used to investigate differences in dependent variables between treatment and control groups at 6 weeks and 2 months.

For BrEQ scores and mastectomy flap edema thickness analysis across multiple time points, a mixed-model analysis of variance (ANOVA) was employed. Time was identified as the within-subjects factor, whereas group assignment (experimental vs control) was the between-subjects factor. When a significant main effect was observed, Bonferroni post hoc tests were conducted to identify significant differences between mean values. Furthermore, the presence of pitting edema was examined through the application of Fisher's exact test.

IBM SPSS Statistics 29 (IBM, Armonk, NY) was used, and statistical significance was determined by 2-tailed *P*-values ≤.05.

## RESULTS

**Figure 2.**
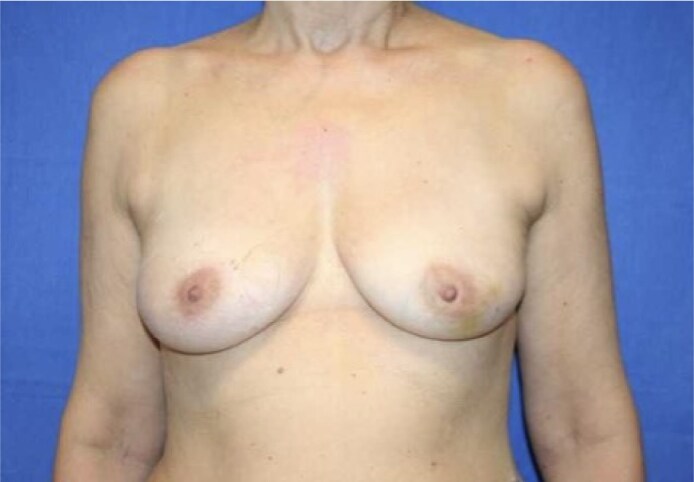
Preoperative visit of an experimental group participant undergoing bilateral total mastectomy and reconstruction with mammary expanders. Female patient, 48 years old.

**Figure 3.**
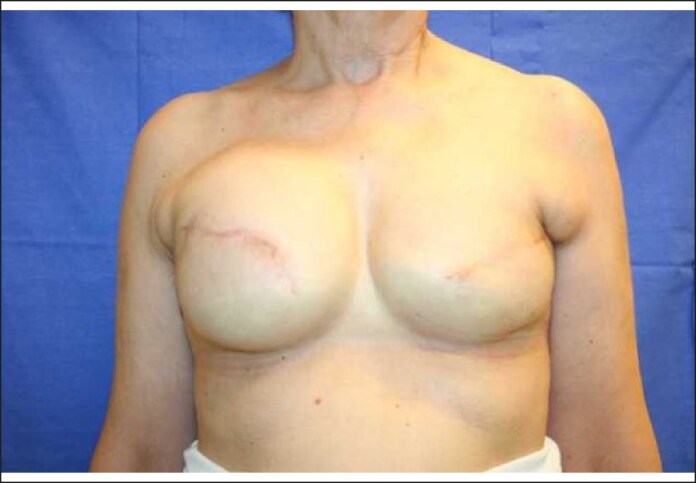
A 6-month postoperative visit of an experimental group participant at the end of the expansion process. Female patient, 48 years old.

Patient demographics, surgical details, and medical histories were recorded at the preadmission visit (Table 1). In this study, a total of 24 patients were enrolled, with 12 individuals assigned to each group. The age range in the control group was 42 to 74 years, with a mean age of 59.17 years, whereas in the experimental group, the age range was 30 to 69 years, with a mean age of 48.33 years. Within this patient cohort, 20 patients (10 per group) underwent unilateral mastectomy, whereas 4 patients (2 per group) underwent bilateral mastectomy, for a total of 28 breasts included in the analysis. Among these, 14 were ascribed to the control group (*n* = 14) which included 10 nipple-sparing mastectomies (71.4%) and 4 total mastectomies (28.6%), and 14 to the experimental group (*n* = 14) with 8 nipple-sparing mastectomies (57.1%) and 6 total mastectomies (42.8%). For each group, 3 patients (25%) underwent direct-to-implant reconstruction after mastectomy (25%), whereas for the remaining 9 patients (75%), a mammary expander was placed (Figures 2, 3).

**Table 1.** Patient's Demographic and Surgical Characteristics

Demographic characteristics	Experimental group	Control group	*P*-value
Age, mean (SD)	48.33 (10.38)	59.17 (11.20)	.001
BMI, mean (SD)	24.38 (6.34)	24.62 (4.30)	.438
Active smokers	5 (41.67%)	5 (41.67%)	.319
Comorbidities			
Cardiovascular	4 (33.33%)	4 (33.33%)	.252
Respiratory	1 (8.33%)	1 (8.33%)	.279
Metabolic	1 (8.33%)	3 (25%)	.163
Adjuvant therapy			
Chemotherapy	3 (25%)	1 (8.33%)	.148
Radiotherapy	3 (25%)	1 (8.33%)	.148
Neoadjuvant therapy	1 (8.33%)	2 (16.67%)	.279
Previous breast surgery (lumpectomy)	3 (25%)	3 (25%)	.5
Bilateral mastectomy	2 (16.67%)	2 (16.67%)	.0325
*Surgical data*			
Side			
Left	8 (66.67%)	4 (33.33%)	.135
Right	4 (33.33%)	8 (66.67%)	.249
Mastectomy type			
Nipple sparing	7 (58.33%)	9 (75%)	.12
Total	5 (41.67%)	3 (25%)	.026
Axillary dissection	4 (33.33%)	3 (25%)	.211
Reconstruction technique			
Direct-to-implant (definitive prosthesis)	3 (25%)	3 (25%)	.338
Two stage (tissue expander)	9 (75%)	9 (75%)	.338
Implant characteristics			
Mentor	9 (75%)	9 (75%)	.279
Polytech	3 (25%)	3 (25%)	.279
Implant volume (cc), mean (SD)	383.21 (78.80)	362.50 (96.19)	.269

SD, standard deviation.

Three patients were unable to successfully complete follow-up (2 patients who underwent unilateral mastectomy in the experimental group and 1 patient who underwent bilateral mastectomy in the control group). Therefore, a total of 24 breasts were analyzed, evenly distributed between the control group (*n* = 12) and the experimental group (*n* = 12). On average, 383.21 g (standard deviation [SD]: 78.80 g) of breast tissue were removed from the experimental group and 362.50 g (SD: 96.19 g) from the control group. All the patients of the experimental arm adhered to the medication regimen throughout the study duration, and no side effects were reported.

Demographic and surgical data exhibited congruent characteristics between the 2 groups, with no statistically significant distinctions (Table 1).

### Primary Outcomes

**Figure 4.**
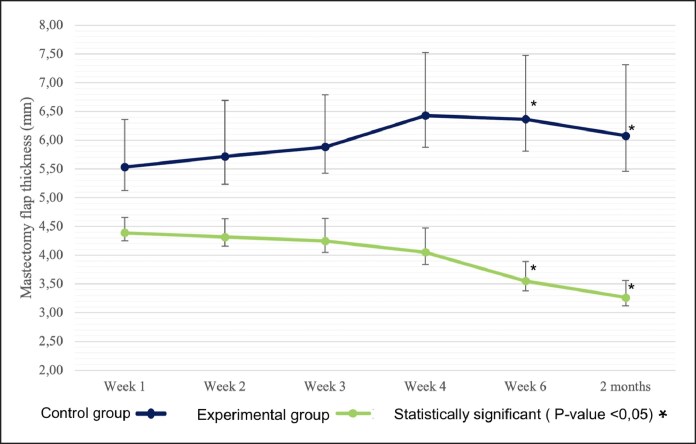
Mean values of mastectomy flap thickness with 95% CI bars during follow-up visits.

The mean measurements of mastectomy edema flap thickness displayed variations throughout the follow-up period. Specifically, in the control group (Table 2, Figure 4), the mean values exhibited an upward trajectory initially, stabilizing during the last 3 assessments at the 4-week, 6-week, and 2-month postoperative visits. Conversely, the experimental group presented a contrasting pattern with mastectomy flap thickness measurements gradually decreasing.

**Table 2.** Mean and Standard Deviation of Mastectomy Flap Thickness

	Experimental group		Control group		Δ Mean (%)	Post hoc comparison (Bonferroni's method) *P*-value
	Mean (mm)	SD	Mean (mm)	SD		
Week 1	4.39	0.51	5.54	1.55	−20.8	.027
Week 2	4.32	0.59	5.72	1.82	−24.6	.011
Week 3	4.25	0.74	5.88	1.70	−27.7	.003
Week 4	4.05	0.79	6.43	2.05	−37.0	<.001
Week 6	3.55	0.64	6.36	2.09	−44.2	<.001
Week 8	3.27	0.51	6.08	2.14	−46.2	<.001

Corresponding post hoc comparisons according to Bonferroni's method. SD, standard deviation.

The experimental group showed a significant reduction in flap thickness at each subsequent assessment (Table 2). Starting from a mean thickness of 4.42 mm at Week 1, the measurements decreased progressively to 3.27 mm by the 2-month evaluation (*P* < .001). This represents a 45.95% decrease in thickness compared with the control group's mean thickness of 6.05 mm at the same time point. The most substantial reductions were observed at the 6-week and 2-month evaluations, with decreases of 44.18% and 45.95%, respectively, highlighting the efficacy of this therapy in reducing postmastectomy edema.

The control group's mean thickness values remained relatively higher throughout the follow-up period, starting at 5.50 mm in Week 1 and showing minimal reductions, stabilizing around 6.05 mm at the 2-month evaluation. These findings underscore the impact of Linfadren on reducing edema, evidenced by the decreasing trend in the experimental group's mastectomy flap thickness measurements over time.

In both the experimental and control groups, paired-sample *t* tests unveiled statistically significant differences in mastectomy flap thickness between baseline and Week 6 (*P* < .001) and 2 months following the operation (*P* < .001; Table 3).

**Table 3.** Paired and Independent *t* Test Comparison in Mastectomy Flap Thickness at 1 Week and 8 Weeks Post Reconstruction

	Experimental group		Control group		Δ Mean (SD)	*t* (df)	*P*-value
	Week 1	Week 8	Week 1	Week 8			
	4.39 (0.51)	3.27 (0.51)	5.54 (1.55)	6.08 (2.14)	2.79 (2.09)	4.39 (12.26)	<.001*
Mean (SD)	1.15 (0.48)		−0.55 (0.76)				
*t* (df)	8.37 (11)		−2.05 (11)				
*P*-value	<.001		.029				

df, degrees of freedom; SD, standard deviation. *Significant value.

To assess disparities in mastectomy flap thickness between control and experimental groups, independent samples *t* tests were carried out (Table 3). These analyses uncovered statistically significant differences between the 2 groups at 6 weeks and 2 months postbreast reconstruction (*P* < .001). These results were adjusted in accordance with Levene's test for variance inequality. The utilization of a mixed-model ANOVA (Table 4) disclosed statistically significant effects of time (*P* = .034) and treatment (*P* = .01) on mastectomy flap thickness. Additionally, a statistically significant interaction between treatment and time was observed (*P* < .001). Subsequent post hoc tests, using Bonferroni's method (Table 2), indicated significant differences between control and experimental groups across all follow-up visits, especially notable at Weeks 4, 6, and 8 (*P* < .01).

**Table 4.** Mixed-ANOVA Results Comparing Group and Time Effects on Mastectomy Flap Thickness

	*F*(df)	*P*-value	*ηp* ^2^
Time effects	*F*(4,104) 2.48	.034	0.087
Group effects	*F*(1,26) 13.19	.001	0.34
Time-group effects	*F*(4,104) 22.68	<.001	0.47

ANOVA, analysis of variance; df, degrees of freedom.

### Secondary Outcomes

**Figure 5.**
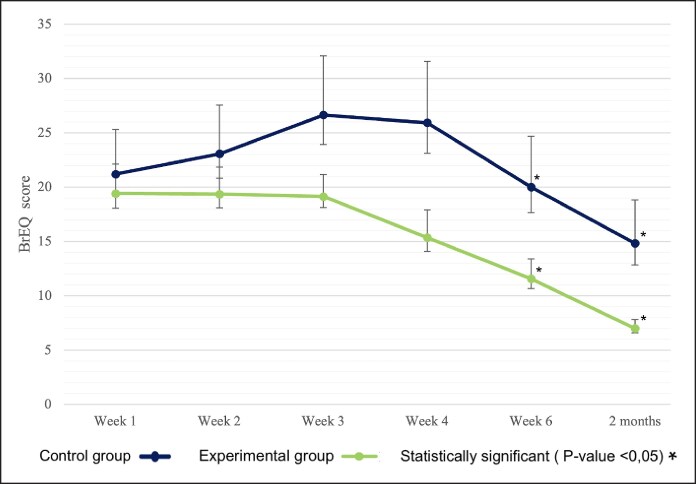
Mean of Breast Edema Questionnaire (BrEQ) total scores with 95% CI bars during follow-up visits.

The highest average total scores on the BrEQ were observed at baseline for the experimental group, whereas the control group reached its peak scores at Week 3. Over time, there was a gradual decline in the average BrEQ scores, much more evident in the experimental arm (Figure 5).

In the experimental group, paired-sample *t* tests revealed a significant difference in BrEQ scores between Week 1 and Week 6 (*P* < .001). Similarly, significant differences were observed when comparing Week 1 scores to those at the 2-month follow-up (*P* < .001). In contrast, paired-sample *t* tests conducted within the control group showed a significant difference only between Week 1 and the 2-month follow-up (*P* = .03; Table 5)

**Table 5.** Paired and Independent *t* Test Comparison in Breast Edema Questionnaire Results at 1 Week and 8 Weeks Post Reconstruction

	Experimental group		Control group		Δ Mean (SD)	*t* (df)	*P-*value
	Week 1	Week 8	Week 1	Week 8			
	19.33 (5.37)	7.00 (1.41)	21.42 (7.13)	14.83 (6.92)	7.83 (6.32)	3.84 (11.96)	.002
Mean (SD)	12.33 (5.33)		6.58 (5.98)				
*t* (df)	8.014 (11)		3.81 (11)				
*P*-value	<.001		.003				

df, degrees of freedom; SD, standard deviation.

Independent samples *t* tests, adjusted in accordance with Levene's test, were employed to analyze differences in BrEQ scores between the control and experimental groups, unveiling significant differences at 6 weeks (*P* = .004) and at 2 months post surgery (*P* = .002).

The mixed-model ANOVA, assessing the impact of time and treatment on BrEQ scores, identified statistically significant effects of time (*P* < .001) and treatment (*P* = .011). A significant interaction between treatment and time was also noted (*P* < .001).

Subsequent post hoc comparisons, conducted using Bonferroni's method, revealed no statistically significant differences between the control and experimental groups during the initial 2 weeks. However, significant differences were detected at Week 3 (*P* = .016) and remained statistically significant at Weeks 4 and 6 (*P* = .002).

**Figure 6.**
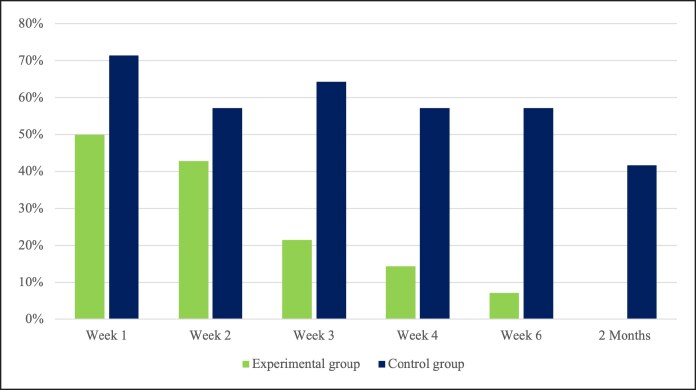
Percentage of patients with pitting edema in experimental and control groups.

With regard to the presence of pitting edema, the percentage of patients exhibiting this condition was notably higher in the control group (58.54%) compared with the experimental group (23.17%). This trend persisted throughout each postoperative ambulatory visit (Figure 6). To assess the overall percentage of patients experiencing pitting edema during the postoperative follow-up, Fisher's exact test was applied. The analysis revealed a statistically significant association between the presence of pitting edema and the absence of anti-edema treatment (*P* < .001).

No significant events, such as infections, were documented in our study. We observed 1 instance of seroma in both treatment and control groups. Additionally, a single case of wound dehiscence was noted in the treatment group, which did not necessitate surgical revision.

## DISCUSSION

This clinical trial underscores the efficacy of Linfadren (Omega Pharma Srl) as a preventive strategy against breast edema in postmastectomy implant-based reconstruction. Patients in the treatment group demonstrated significant improvements in both mastectomy flap thickness measurements and BrEQ scores. Specifically, as shown in Table 2, the experimental group experienced a notable reduction in mastectomy flap thickness over time, with a 45.95% decrease by the 2-month mark, compared with the control group's stable or increasing thickness values. The control group exhibited a gradual increase in mastectomy flap thickness, emphasizing the lack of effective treatment. The significant differences between the groups at 6 weeks and 2 months (Table 2) highlight the effectiveness of a compound based on diosmin, coumarin, and arbutin in reducing edema. Although the control group showed a progressive increase in flap thickness, the experimental group exhibited a substantial decrease; pitting edema was more prevalent in the control group throughout the study, with the number of positive assessments decreasing over time but still consistently higher than in the treatment group.

This aligns with the understanding that lymphedema can present without pitting edema because of tissue induration and fibrosis, typically seen in chronic rather than acute edema.^20^ These primary findings support the role of diosmin, coumarin, and arbutin in mitigating early postoperative edema.

BrEQ scores for the control group were higher on average than those of the experimental group, with an initial upward trend in the first few weeks post operation. However, both groups showed improvement in symptoms from 3 weeks post operation, when patients typically begin regaining sensation in the operated breast(s). The experimental group displayed a stable trend in BrEQ scores during the initial weeks, followed by gradual improvement over time, likely because of Linfadren's (Omega Pharma Srl) preventive action against edema formation and symptom alleviation.

Conservative treatment approaches for lymphedema, particularly concerning breast edema, entail substantial costs and time commitments.^21^ Previous research already established the effectiveness of benzopyrones such as diosmin in the treatment of lymphedema.^16,22-27^

The formulation employed already demonstrated its effectiveness in addressing secondary upper limb lymphedema following breast cancer surgery.^16^ These compounds promote fluid drainage and enhance proteolysis through macrophages, reducing excess interstitial proteins; their administration leads to a sustained reduction in lymphedema, ameliorating the associated inflammatory conditions.^23^ Breast edema may delay the expansion process, lengthening the reconstructive process and potentially causing psychological distress.^28^ Moreover, it can complicate the assessment of breast volume, presenting challenges in evaluating outcomes and surgical planning.^29^ Left untreated, this condition often persists, leading to delays in care, higher healthcare costs, and suboptimal aesthetic outcomes.^3,30,31^

Introducing an effective anti-edema treatment improves patient outcomes and reduces long-term costs by minimizing chronic therapy. Notably, no existing literature has explored benzopyrones for breast edema specifically. Previous studies focused on upper limb lymphedema, limiting direct comparisons, but our findings align with the general effectiveness of benzopyrones in lymphedema management.^16,23,32^ These findings are further supported by pharmacogenomic studies.^22^

This study has several limitations. Firstly, it involved a small number of enrolled patients. Given the scarcity of data, it was not feasible to estimate the required sample size during the study's planning phase. However, for tightly controlled experimental research, successful results can still be achieved even with relatively small sample sizes.^33^ Secondly, the relatively short duration of the study precluded the assessment of long-term outcomes. In this study, the authors did not employ blinding procedures, which heightens the risk of bias in result interpretation. Additionally, baseline measurements of mastectomy flap thickness were taken directly at Week 1, after therapy had already commenced. Indeed, lack of such measurement before the medication was started is a major limitation, yet immediate postoperative results may not be reliable as they precede the development of edema. The measurement of flap thickening in this study was conducted at a specific point, typically the inferior pole of the breast, because this area is usually subjected to a higher degree of both internal and external stress because of the relatively limited soft tissue available to support an implant. The influence of gravity is also significant in tissue fluid dynamics, contributing to heightened capillary pressure and creating impediments to the transportation of lymph fluid against gravity. Furthermore, gravity directly facilitates the downward movement of fluid through the interstitium.^34^ However, it should be acknowledged that breast edema can manifest in other quadrants of the breast without affecting the remaining breast tissue. Such a limit may be overcome by measuring flap thickness at multiple points to calculate an average flap thickness. Vernier caliper measurements are subject to operator-dependent variation. Techniques such as ultrasound might offer valuable insights. However, performing these measures on a weekly basis during the initial evaluation may not be practical.

In this study, the authors provide a framework for future research on breast edema prophylaxis, including applications in patients receiving breast radiotherapy.

## CONCLUSIONS

The significant differences observed between and within the groups suggest that anti-edema formulations may have a role as prophylactic treatments in postmastectomy implant-based reconstruction. Our findings indicate that Linfadren (Omega Pharma Srl) may contribute to a reduction in mastectomy flap thickness and an improvement in postoperative outcomes. The observed decrease in flap thickness and the lower prevalence of pitting edema in the treatment group support the potential of diosmin, coumarin, and arbutin in managing postmastectomy edema.
